# A Rapid Culture Method for the Detection of *Campylobacter* from Water Environments

**DOI:** 10.3390/ijerph18116098

**Published:** 2021-06-05

**Authors:** Nicol Strakova, Kristyna Korena, Tereza Gelbicova, Pavel Kulich, Renata Karpiskova

**Affiliations:** Veterinary Research Institute, 621 00 Brno-Medlánky, Czech Republic; korena@vri.cz (K.K.); gelbicova@vri.cz (T.G.); kulich@vri.cz (P.K.); karpiskova@vri.cz (R.K.)

**Keywords:** centrifugation, wastewater, surface water, *Campylobacter*, culture method, filtration

## Abstract

The natural environment and water are among the sources of *Campylobacter jejuni* and *Campylobacter coli*. A limited number of protocols exist for the isolation of campylobacters in poorly filterable water. Therefore, the goal of our work was to find a more efficient method of *Campylobacter* isolation and detection from wastewater and surface water than the ISO standard. In the novel rapid culture method presented here, samples are centrifuged at high speed, and the resuspended pellet is inoculated on a filter, which is placed on *Campylobacter* selective mCCDA agar. The motile bacteria pass through the filter pores, and mCCDA agar suppresses the growth of background microbiota on behalf of campylobacters. This culture-based method is more efficient for the detection and isolation of *Campylobacter jejuni* and *Campylobacter coli* from poorly filterable water than the ISO 17995 standard. It also is less time-consuming, taking only 72 h and comprising three steps, while the ISO standard method requires five or six steps and 144–192 h. This novel culture method, based on high-speed centrifugation, bacterial motility, and selective cultivation conditions, can be used for the detection and isolation of various bacteria from water samples.

## 1. Introduction

The genus *Campylobacter* comprises 33 species. These are Gram-negative, microaerophilic, motile, curved or spiral-shaped bacteria [1]. Thermophilic species, such as *Campylobacter *jejuni** and *Campylobacter *coli**, are the most frequently reported ones associated with human gastrointestinal infections [2,3,4]. 

Campylobacteriosis is a leading foodborne gastroenteritis of bacterial origin in Europe [2]. The course of the disease is characterised by nausea, fever, abdominal cramps, watery to bloody diarrhoea, and vomiting [3]. In severe cases, campylobacteriosis is associated with serious postinfectious complications, such as peripheral nervous system damage, Guillain–Barré syndrome, and reactive arthritis [5,6]. 

*C. jejuni* and *C. coli* are frequently detected as commensal bacteria in chickens [2,7,8]. Despite the fact that poultry is considered to be a major source of campylobacter infections in humans, multiple other sources exist, including other domestic and wild animals and wild birds [9,10,11]. Recently, it has been reported that agricultural waste used in waste-to-energy processes is an important source of campylobacters [12]. Faeces of wildlife, such as deer and other ruminants, may also act as a source of campylobacters in the environment [9]. Waterborne *Campylobacter* spp. are assumed to originate from animal faeces, agricultural leaks, and wastewater contamination. *C. jejuni* and *C. coli* are often present in aquatic environments, which may provide a further source of infections [13,14]. Several studies have shown that campylobacters can also be detected in drinking water [15,16,17] and in untreated wastewater [13,14,18]. Bacteria such as *C. jejuni* and *C. coli* can be released by wastewater treatment plants into the environment [19]. Exposure to surface water for leisure activities and consumption of unwashed raw fruits and vegetables that have been irrigated or in contact with contaminated water also pose potential risks [20,21]. Therefore, detecting thermotolerant campylobacters in water samples is an important task for public health professionals. 

There are only a limited number of protocols for *Campylobacter* detection from turbid water samples. Stagnant turbid water can often contain organic pollution (leaves, mud, water algae, protozoa, wood pieces, etc.), and the filtration process becomes rather complicated in actual practice, as sedimenting or floating impurities are unsuitable for filtration [22]. ISO standard 17995, a protocol from 2005 [23], was only applicable to filterable water. The standard was modified in 2019 and is suitable for all water types now. The ISO standard declares that users who intend to employ this method are expected to verify its performance for the particular matrix under their own laboratory conditions. Therefore, we used filters with a smaller pore size (0.22 μm) for more effective “catching” of campylobacters from water environments. We supposed that filtration using higher pressure on cells would allow *C. jejuni* or *C. coli* to easily pass through filters with a pore size of 0.45 μm, especially bacteria with a size of 0.2–0.45 μm. Moreover, a prefiltration step with a 1.4 µm filter was added for removal of organic pollution. The goal of our work was to develop a universal method for the isolation of thermotolerant campylobacters in turbid water that can supplement the ISO standard. It is well known that campylobacters in water can be detected by noncultivation methods that exhibit more rapid and accurate detection, but they have disadvantages, such as the further possibility of strain characterisation (e.g., determination of MLST, antibiogram, whole-genome sequencing, and further analysis for epidemiological purposes) and no discrimination between viable and nonviable cells [24,25]. 

Therefore, this paper presents a rapid, easy, and effective cultivation-based approach suitable for the isolation of thermotolerant campylobacters from the surface water of ponds and lakes in nature and from municipal wastewater treatment plants. 

## 2. Materials and Methods

### 2.1. Sample Collection

One litre of water was collected from 20–50 cm depth by submerging the bottle upside down, followed by slow rotation under the water level until the bottle was filled, using a telescopic sampling stick. In total, 36 water samples were collected for parallel detection of thermotolerant campylobacters by 2 methods. The samples were collected during all seasons of the year to cover year-round conditions. Immediately after sampling, the samples were transferred to the laboratory in a cooling box. In total, 11 samples originated from the final output of municipal wastewater treatment plants and 25 surface water samples were from aquatic environments, mainly ponds and lakes in 17 localities in the Czech Republic. Water samples were taken according to the EN ISO 19458 standard procedure [26]. 

### 2.2. Isolation of Campylobacters from Poorly Filterable Water Samples by the Standard Cultivation Method

ISO 17995:2019 was used as a reference method for the isolation of campylobacters from wastewater and surface water samples [27]. Briefly, water samples (500 mL) were prefiltered (1.4 μm glass filter; Duren, Macherey Nagel, Germany) for quick removal of mechanical impurities and filtered (0.22 μm, mixed cellulose ester filter; Merk, Darmstadt, Germany); the filters were then transferred into 2 campylobacter selective broths (Preston and Bolton broth) for enrichment and incubated at 42 °C in an anaerostat (AnaeroJar, Oxoid, Basingstoke, UK) under a microaerobic atmosphere (CampyGen 3.5 L, Oxoid, Basingstoke, UK). After 44 ± 4 h of incubation, the inoculum was cultivated on *Campylobacter* blood-free selective agar (modified charcoal–cefoperazone–deoxycholate agar (mCCDA), Oxoid, Basingstoke, UK) and incubated for another 44 ± 4 h. After incubation under a microaerobic atmosphere at 42 °C, isolation of presumptive colonies on nonselective agar (blood agar) and mCCDA was performed under the same conditions. Finally, *Campylobacter* spp. were identified (see below). 

### 2.3. Novel Cultivation Method for Campylobacters from Poorly Filterable Water Samples

In parallel, *Campylobacter* spp. were isolated from water samples by a novel method without enrichment. In detail, 50 mL water samples were centrifuged at 12,000× *g* (acceleration time 300 s, deceleration time 40 s; HERMLE Z326K, Wehingen, Germany) for 30 min at 10 °C. Pellets were resuspended in 200 μL of sterile water and spread on a filter (0.45 μm, mixed cellulose ester filter; Merk, Darmstadt, Germany). Filters were placed face-up on top of mCCDA agar and incubated bottom-down for passive filtration at 42 °C under a microaerobic atmosphere. After overnight incubation, the filters were removed and cultivation continued at 42 °C under a microaerobic atmosphere for another 44 ± 4 h. Experiments were performed in triplicate. 

### 2.4. Campylobacter Species Identification

Suspected *C. jejuni* and *C. coli* colonies cultivated on mCCDA were plated on blood agar at 42 °C under a microaerobic atmosphere for 44 ± 4 h, and bacterial DNA was extracted by boiling. Multiplex PCR using PPP master mix (Top-Bio, Vestec, Czech Republic) was used to identify the genus *Campylobacter* and 2 species, *C. jejuni* and *C. coli*. The primers used (Generi-Biotech, Hradec Kralove, Czech Republic) [24,25,28] are shown in Table S1. Amplification was carried out with the following PCR conditions: 95 °C for 5 min, 35 cycles at 94 °C for 30 s, 55 °C for 30 s and 72 °C for 1 min, and 72 °C for 7 min. 

Matrix-assisted laser desorption/ionisation time-of-flight mass spectrometry (MALDI-TOF/MS) was used for confirmation of *Campylobacter* species. In detail, when pure bacterial cultures were obtained, MALDI-TOF/MS with ethanol and formic acid extraction was used, and isolates were identified after comparing the bacterial spectrum in a Bruker database MBT 8468 by Biotyper software (version 3.1, Bruker Daltonics GmbH, Bremen, Germany). 

### 2.5. Campylobacter Cell Numbers

Cell numbers of *C. jejuni* and *C. coli* were compared before and after centrifugation. Briefly, the bacterial suspension was prepared in 50 mL MilliQ water with optical density OD_600nm_ 0.8–1.2. The suspension was centrifuged at 12,000× *g* for 30 min, the supernatant was removed, and the pellet was diluted in 50 mL MilliQ water. The cell number was evaluated by the optical density OD_600nm_ before and after centrifugation at high speed. 

### 2.6. Campylobacter Viability

The viability of campylobacters after centrifugation at high speed was tested on randomly selected *C. jejuni* and *C. coli* strains isolated from the water environment. The viability of bacterial cells was tested by colony enumeration of *C. jejuni* and *C. coli* after growth on agar plates. Ten-fold dilution series of bacterial suspension (100 μL) were applied on the mCCDA and chromogenic Brilliance CampyCount Agar (Oxoid, Perth, UK), and cultivation was performed for 24 ± 4 h at 42 °C under a microaerobic atmosphere. Finally, the numbers of colonies on the plates were evaluated before and after centrifugation.

### 2.7. Campylobacter Morphology

Transmission electron microscopy with negative staining of samples was performed for *Campylobacter* morphology testing. Briefly, randomly selected bacterial strains were suspended in MilliQ water. The suspension was covered by a grid-coated formvar film (Merk, Darmstadt, Germany) and carbon (Agar Scientific, Essex, UK) for 5 min, and the residual water was dried. Finally, 2% aqueous phosphotungstic acid was placed on the grid, and the sections were observed under a Philips 208 S Morgagni electron microscope (FEI, Dresden, Germany) with magnification of 14,000–36,000× and accelerating voltage of 80 kV.

### 2.8. Campylobacter Motility 

Swarming soft agar motility assay was used to observe *Campylobacter* motility phenotypes [29,30]. The bacterial suspension (10 μL) was placed on 0.25% Lab Lemco agar (Oxoid, Basingstoke, UK). The cultivation was performed for 24 ± 4 h at 42 °C under a microaerobic atmosphere. Finally, the bacterial motility of *C. jejuni* and *C. coli* was compared before and after centrifugation. Non-motile *Klebsiella pneumoniae* were used as negative control. 

### 2.9. Evaluation of Selectivity of the Novel Culture-Based Method

To check the selectivity of the novel culture-based method, non-motile *Klebsiella pneumoniae* were mixed with *C. jejuni* and *C. coli.* The tested bacterial suspensions (200 μL) were spread on filters (0.45 μm, mixed cellulose ester filter; Merk, Darmstadt, IN, USA) and incubated on mCCDA or Brilliance CampyCount Agar. Petri dishes were incubated bottom-down at 42 °C for 24 ± 4 h under a microaerobic atmosphere. After overnight incubation, the filters were removed, the dishes were inverted, and cultivation continued at 42 °C under a microaerobic atmosphere for another 44 ± 4 h. Finally, bacterial species were identified by PCR and MALDI-TOF/MS. Experiments were performed in triplicate.

### 2.10. Measurement of Diagnostic Accuracy of the Two Methods

A comparison of the modified detection method and the standard ISO method according to diagnostic accuracy was conducted according to EN ISO 16140–4:2020, commonly used as a validation method in food chain microbiology. This protocol is used for verification of reference methods and validated alternative methods in a single laboratory [31]. Briefly, the diagnostic accuracy, relative sensitivity, and relative specificity were calculated using the following formulas:

diagnostic accuracy (%) = (A + D)/(A + B + C + D) × 100

relative sensitivity (%) = A/(A + B) × 100

relative specificity (%) = D/(C + D) × 100

where A is the number of positive samples by both methods, B is the number of positive samples by the standard method, C is the number of positive samples by the modified method, and D is the number of negative samples by both methods.

## 3. Results

### 3.1. Detection of Campylobacter in Poorly Filterable Water Samples 

**Figure 1:**
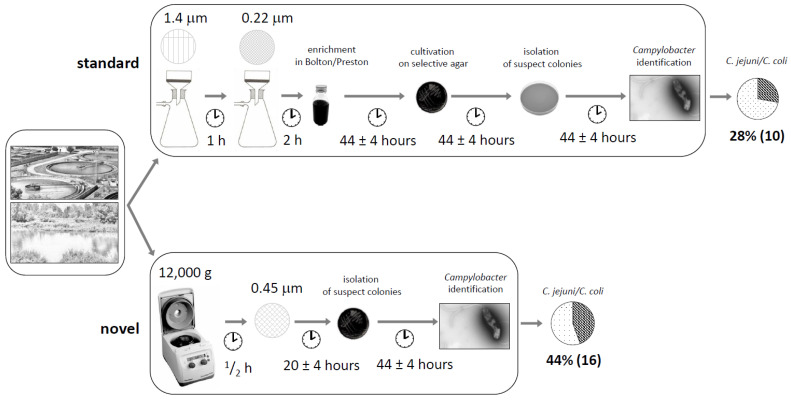
Scheme of standard and novel rapid method for detection of campylobacters, including timing and effectiveness. ISO standard method (above) and novel method (below) were used for isolation and detection of campylobacters from wastewater and surface water samples. Positive samples shown in dark colours.

Illustrations of the two methods for the detection of *Campylobacter* in water samples are shown in detail in Figure 1. The scheme of the standard method with slight modifications is shown in the upper part of the figure. The presented novel method (lower part of the figure) was based on the presumption that campylobacters are motile and can pass through filter pores. Campylobacter selective agar mCCDA was used to suppress the growth of redundant bacterial species in the water environment. The protocols are described above in detail. The rapid alternative method was easier to perform and took less time, saving approximately 72 h. *Campylobacter* identification was easier due to lower background microbiota levels of non-motile bacterial species. A water volume sample as small as 50 mL was a sufficient default volume for the isolation of *Campylobacter* by the modified method (Figure 1). 

### 3.2. Comparison of Effectiveness between Standard and Modified Methods 

The two methods used for the detection of thermotolerant campylobacters in water samples without distinguishing between water sources were compared (Figure 1). Of the 36 samples examined, 10 (28%) were positive for *Campylobacter* spp. according to the standard ISO method and 16 (44%) samples according to the modified method (Figure S1). In total, the effectiveness of thermotolerant *Campylobacter* strain detection from all water samples was 1.5 times higher with the modified method than the standard method (Figure 1). A list of all samples with detected and isolated *Campylobacter* strains is presented in Table S2.

Detection of campylobacters by the standard method was performed after enrichment in two selective broths, Bolton and Preston broth. Bolton broth was more efficient (22%; 8/36) for enrichment of *Campylobacter* from water samples than Preston broth (14%; 5/36). The presented novel culture-based method failed only in one sample compared with the standard method, which failed six times (Table S2). A statistical analysis of diagnostic accuracy was performed. The relative sensitivity and specificity were calculated. The relative sensitivity and specificity of the modified method were 90.0% and 73.08%, respectively. The diagnostic accuracy of the method was 77.78% (Table S3). 

**Figure 2:**
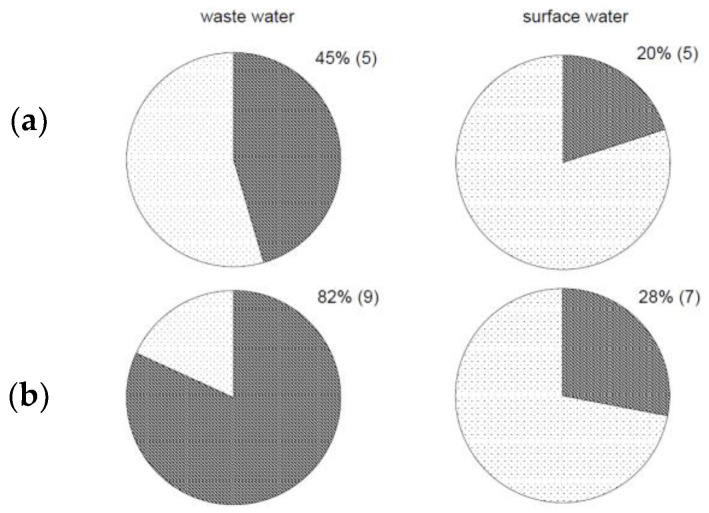
Comparison of detection of thermotolerant campylobacters from water samples. Percentage (number) of *Campylobacter*-positive samples from wastewater (left charts) and surface water (right charts). Isolation and detection of *C. jejuni* and *C. coli* by (**a**) standard ISO 17995 method and (**b**) alternative method. Positive samples shown in dark colour.

The detection of thermotolerant campylobacter strains was compared between the two methods based on water type (Figure 2); 45% of wastewater samples and 20% of surface water samples were positive (Figure 2a) according to the standard method with enrichment. According to the modified method, 82% of wastewater samples and 28% of surface water samples were positive for thermotolerant campylobacters (Figure 2b). 

**Figure 3:**
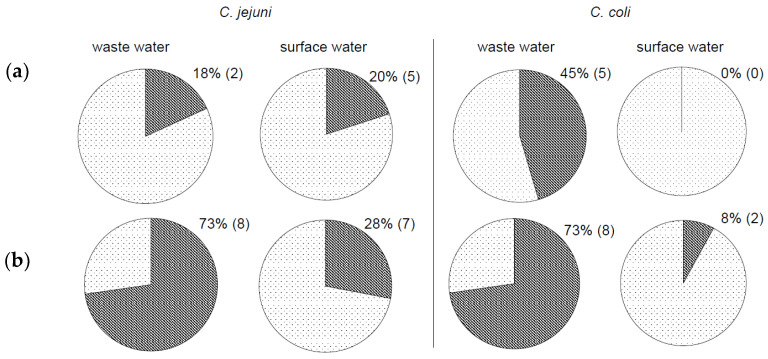
Comparison of isolation of thermotolerant *C. jejuni* and *C. coli* from water samples. Isolation of *C. jejuni* by (**a**) standard ISO 17995 method and (**b**) culture-based method without enrichment. Positive samples shown in dark colour.

More details on the isolated campylobacter species are shown in Figure 3. From each plate, we identified more than one bacterial colony, so the number of *Campylobacter* isolates (Figure 3) was higher than the number of positive water samples (Figure 1). Surface water and, even more, wastewater contained a mixture of bacteria, and isolation of campylobacters from the water microbiota was difficult. However, we can summarise that the novel alternative method was more efficient at detecting and isolating thermotolerant *Campylobacter* strains in both wastewater and surface water. Our results show that campylobacters isolated by the culture-based method without enrichment were more easily obtained from agar media. Moreover, the method put neither *C. jejuni* nor *C. coli* at a disadvantage (Figure 3b).

### 3.3. Evaluation of the Novel Method 

**Figure 4:**
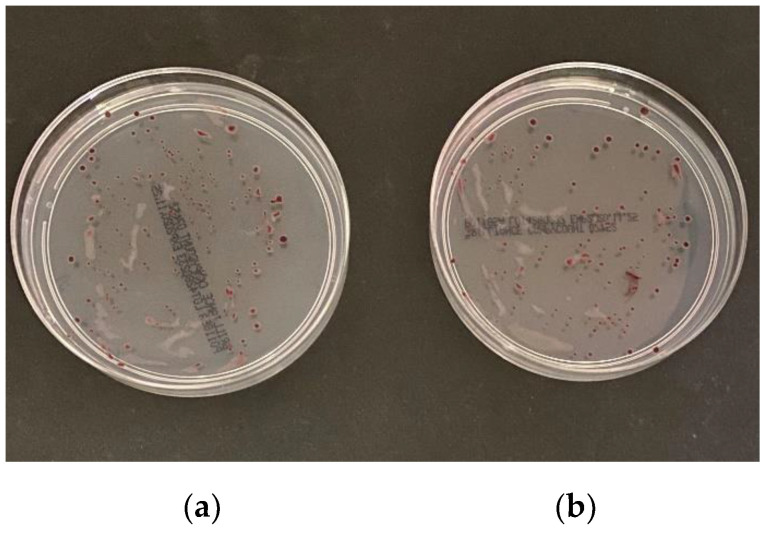
Campylobacter viability. *C. jejuni* strain was cultivated on Brilliance CampyCount Agar (**a**) before and (**b**) after centrifugation.

An evaluation of the novel method was performed by comparing the cell numbers, viability, morphology, and motility of *Campylobacter* spp. to confirm its suitability for *Campylobacter* spp. isolation. First, we checked whether high-speed centrifugation at 12,000× *g* would harm *Campylobacter* spp. and changed the cell numbers. Our results show that the cell numbers did not change, because OD_600nm_ of bacterial suspensions was similar before and after centrifugation, at 0.38 and 0.34, respectively. Centrifugation has no effect on the viability of *Campylobacter* spp. Our results show that CFU/mL before (1.23 × 10^6^) and after (1.17 × 10^6^) centrifugation remained within the same logarithmic order on Brilliance CampyCount Agar (Figure 4) and on mCCDA (Figure S2, Table S4). 

**Figure 5:**
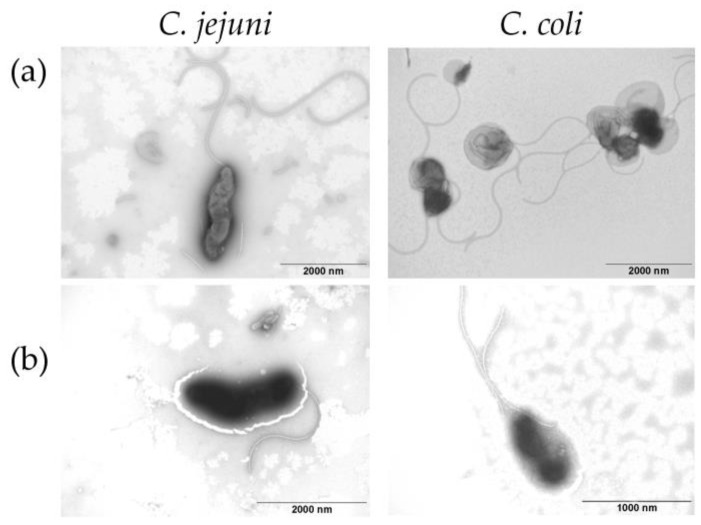
Effect of high-speed centrifugation on *C. jejuni* and *C. coli* cell morphology. Representative figures of electron microscopy (**a**) before and (**b**) after centrifugation. Magnification 14,000×.

Transmission electron microscopy was used for the detection of cell morphology, e.g., flagellas of *C. jejuni* and *C. coli* before and after high-speed centrifugation (Figure 5). Our results show that 12,000× *g* did not damage flagellas in *C. jejuni* and *C. coli*; therefore, they stayed motile without morphologic changes despite high-speed centrifugation. 

**Figure 6:**
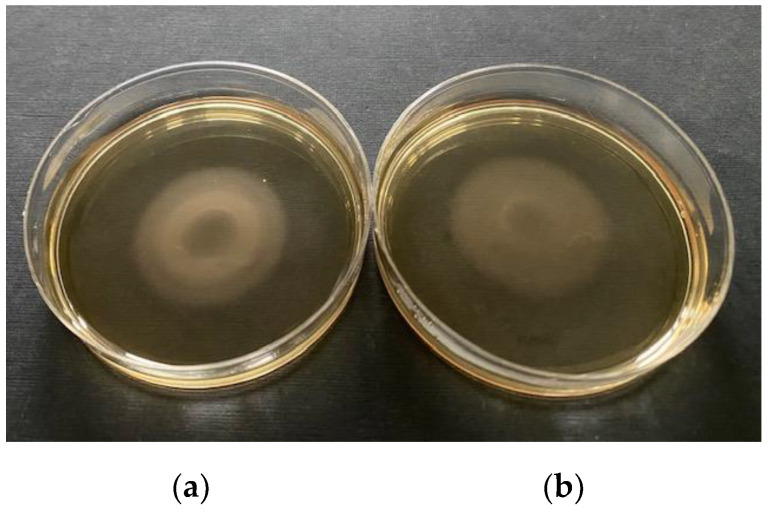
Campylobacter motility. Motility of *C. jejuni* strain did not change (**a**) before and (**b**) after centrifugation.

Finally, the motility assay confirmed that centrifugation had no effect on *C. jejuni* and *C. coli* cell motility; they were both motile before and after centrifugation (Figure 6 and Figure S3). The motility assay together with cell number analysis, cell viability, and cell morphology support the conclusion that centrifugation has no effect on *C. jejuni* and *C. coli*. In summary, our results confirm that high-speed centrifugation does not harm bacterial cells.

The selectivity of the novel method was confirmed by a model mixture of *C. jejuni, C. coli*, and *K. pneumoniae*. Non-motile *K. pneumoniae* (Figures S3 and S4) remained on the filter surface and were removed with the filter after overnight incubation. *C. jejuni* and *C. coli* passed through the pores of the filter and grew well on mCCDA. Species identification was confirmed by PCR and MALDI-TOF/MS (Figures S5 and S6). The *Campylobacter* selective agars contain antibiotics that suppressed the growth of *K. pneumoniae.* The growth of two randomly selected *K. pneumoniae* strains was checked on two *Campylobacter* selective agars, mCCDA and Brilliance CampyCount Agar. These agars allowed weak growth of *K. pneumoniae* as model bacteria of other contaminating bacteria of water samples (data not shown). 

High-speed centrifugation together with the active motion of *C. jejuni* and *C. coli* through 0.45 μm filters did not harm the viability of these bacteria but increased the purity of the target bacteria grown.

## 4. Discussion

The natural environment, including surface waters, agricultural effluent, and marine environments, can be sources of campylobacters for humans [20,32,33,34]. Regarding a low infectious dose (<10^3^ CFU) [35], human exposure can occur not only by the ingestion of food but also by the accidental ingestion of untreated surface water, e.g., during recreational swimming or leisure activities in natural lakes and rivers [36]. Surface water plays an important role in the spread of campylobacteriosis and, together with livestock, pet, and wild animal reservoirs, represents a significant risk to public health [26,37]. The aquatic environment thus may act as a reservoir of campylobacters that are generally considered sensitive to the external conditions of the water environment. Wastewater quality affects the estimation of the fate of pathogenic bacteria discharged from wastewater treatment plants and the risk they pose from the aquatic environment [38]. The next difficulty is determining *Campylobacter*’s sensitivity to stress during laboratory work requiring highly specific conditions for in vitro cultivation, including microaerobic conditions, temperature ranging from 37 to 42 °C, and high water activity. 

Pathogens in urban water are commonly monitored by cultivation and non-cultivation methods [39]. ISO standard 17995 is a cultivation-based gold standard for detection of campylobacters. This standard protocol from 2005 [23] is only applicable to filterable waters. Water is not always suitable for filtration in the external environment because mechanical impurities can clog the pores in the filters, and microbiological analysis of such water becomes extremely difficult when using filtration. The novelisation of the ISO standard in 2019 extended the protocol for all water types by inoculation of non-filterable water sediments in a suitable diluent or directly into enrichment broth [27]. Despite the filtration of low contaminated water samples such as drinking water, which has no problem with clogging of pores, the ISO standard can be replaced by dead-end ultrafiltration because of the pathogen concentration in high water volumes [40].

The noncultivation methods are the second approach for the detection of bacteria [41]. The use of next-generation sequencing (NGS) methods in combination with amplification methods such as qPCR offers rapid and sensitive *Campylobacter* detection in environmental samples [42]. Full-length 16S rRNA gene sequencing can provide identification on the genus and species levels [19]. Unfortunately, noncultivation methods have several disadvantages compared with traditional culture methods. Detection of *Campylobacter* by qPCR can include viable but nonculturable (VBNC) cells or dead cells, or even free DNA, in the water samples. VBNC bacteria remain viable and express various degrees of metabolic activity. *Campylobacter* readily form VBNC cells in water under stress conditions, e.g., low temperature, high pH or high osmolality, and it has been shown that these cells can remain in water for weeks [43]. Other disadvantages include the cost involved, the specialised equipment required, and the expertise of the laboratory technical staff. The detection and isolation of *C. jejuni* and *C. coli* using culture-based methods are complex and time-consuming [44], as our also work confirmed. The presented cultivation method takes only 72 h, while the ISO standard method requires 144–192 h.

The ISO standard cultivation method uses filtration and *Campylobacter* enrichment in selective broths. This method and other modifications use filters with a pore size of 0.45 μm [27,45], which is larger than the campylobacter size (0.2–0.9 μm × 0.5–5 μm), and we supposed a loss of some *Campylobacter* by filtration. Compared to the ISO standard, the rapid culture method uses centrifugation of 50 mL samples and transition of the bacteria through filters. The volume can be increased for the isolation of *Campylobacter* from better quality water samples. Despite the increased volume, the loads stay very low. Our results show that centrifugation without *Campylobacter* enrichment is an effective approach for detection and isolation of *C. jejuni* and *C. coli*. This is in agreement with another study that used centrifugation for the detection of *H. pylori* from drinking water samples [46]. Moreover, high-speed centrifugation allows for smaller water volume and greater efficiency than were reported in that study. High-speed centrifugation can reduce the viability of pathogens [47], so verifications were added to confirm the robustness of the alternative method. It was verified that high-speed centrifugation as used in the modified method has no effect on the number, viability, morphology, or motility of *C. jejuni* and *C. coli* cells. Moreover, this approach can help to separate campylobacters from other bacteria and isolate them even from highly polluted water samples by the passage of *C. jejuni* and *C. coli* through filters. This method used filters with a pore size of 0.45 µm for the active transfer of motile bacteria. Filters with a smaller pore size (0.22 μm) are inappropriate, because campylobacters are bigger and not able to pass through. In general, our modified method can also be used for the detection and isolation of other bacterial species from water samples, but the viability has to be verified. 

The probability of capturing *Campylobacter* depends on the sample volume; 100 mL of water for filtering is recommended to detect 10–100 CFU [27]. As it was supposed that a higher volume would increase the detection rate, the water volume was increased from 100 to 500 mL. Despite the low number of samples, our study clearly shows that the ISO standard discovered fewer positive samples (28%) than the modified method (44%). Moreover, the volume of the ISO standard method was increased. A comparison of the modified and standard ISO methods by diagnostic accuracy, relative sensitivity, and relative specificity was carried out according to EN ISO 16140 [31]. The diagnostic accuracy took into account target and nontarget microorganisms in the presence of a biological matrix, comprised sensitivity and specificity, and was 78%. Relative sensitivity was defined as the ability of the modified method to detect the agent compared to the reference method [27] in the presence of a biological matrix. The relative sensitivity of the modified method was 90%. This parameter can reveal false negative samples. The relative specificity was defined as the ability of the modified method to not detect the target organism when it was not detected by the reference method [48]. This parameter helped to reveal false positive samples, and the relative specificity of the modified method was 73%. Calculating it the other way round, the relative specificity of the standard ISO compared to the modified method is 95%. 

Both methods were also compared on the basis of labour intensity and time consumption. The modified method is easy to perform and offers cleaner bacterial cultures compared to the standard method. When the standard method was used, *Campylobacter* enrichment and isolation on mCCDA agar allowed the survival of a wide spectrum of bacteria; therefore, another subcultivation was needed before the identification step. Our results show that suppression of water microbiota by *Campylobacter* selective agar is a very important task. This is in agreement with another study that used a method to support the growth of *C. jejuni* on *Campylobacter* selective agar, but unlike our approach, those authors filtrated the water samples [45]. 

In our study, we used water samples collected from different localities during all seasons to show the universality of the modified alternative protocol for samples from ponds, lakes, and wastewater treatment plants. The method was not only more sensitive for *C. jejuni* and *C. coli* detection, but also faster and simpler to perform than the ISO method.

## 5. Conclusions

Thermotolerant campylobacters *C. jejuni* and *C. coli* are present in aquatic environments, which may thus represent a source of *Campylobacter* infection. This paper presents a new methodological approach for the detection and cultivation of *C. jejuni* and *C. coli* from wastewater and surface water, without the influence of mechanical impurities of water samples. The modified method was more efficient in detecting thermotolerant campylobacters: 44% samples were *Campylobacter* positive with the modified method and 28% with the standard ISO method. We can conclude that the presented cultivated method was more efficient, faster, smarter, and easier than the ISO method. This method helped to increase the capture of *C. jejuni* and *C. coli* from a turbid aquatic environment; therefore, it can meet the ISO standard guideline.

## Data Availability

All treated data that support the findings of this study are included in the present article. Raw datasets can be obtained from the corresponding author upon request.

## Supplementary Materials

The following are available online at https://www.mdpi.com/article/10.3390/ijerph18116098/s1, Figure S1. Comparison of effective methods for isolation of thermotolerant campylobacters from water samples: (a) ISO standard method and (b) novel method. Level of *Campylobacter* detection in positive samples (*n* = 17) identified by both methods shown in dark colour. Figure S2. Viability testing of *C. jejuni* and *C. coli* on mCCDA agar (a) before and (b) after centrifugation. Figure S3. *Campylobacter* motility. *C. coli* and non-motile *K. pneumoniae* strains were cultivated on 0.25% agar (a) before and (b) after centrifugation. Figure S4. Effect of high-speed centrifugation on *K. pneumoniae* cell morphology. Representative figures of electron microscopy (a) before and (b) after centrifugation. Figure S5. Growth of mixture of thermotolerant campylobacters *C. jejuni* and *C. coli* with *K. pneumoniae* on mCCDA agar. Figure S6. Identification of *C. jejuni* and *C. coli* by PCR and MALDI-TOF/MS after their isolation from mixture of *C. jejuni*, *C. coli*, and *K. pneumoniae*. (a) PCR: size marker (lane 1); mixture of *C. jejuni*, *C. coli*, and *K. pneumoniae* (lane 2); *C. jejuni*-positive control (lane 3); *C. coli*-positive control (lane 4); negative control (lane 5). (b) MALDI-TOF/MS identification. Table S1. Primers used in this study for identification of thermotolerant *C. jejuni* and *C. coli*. Table S2. List of water samples. BW, wastewater; BP, pond/surface water. Localities of ponds where surface water was collected (1–25). Isolation of *C. jejuni* and *C. coli* strains from mCCDA agar. Table S3. Comparison of both methods by diagnostic accuracy (%), relative sensitivity (%), and relative specificity (%). Table S4. Colony formation of *C. jejuni* and *C. coli* before and after centrifugation at 12,000× *g* for 30 min.
