# Supplementary material for: A Rapid Culture Method for the Detection of Campylobacter from Water Environments
Source: Int J Environ Res Public Health. 2021 Jun 5;18(11):6098. doi: 10.3390/ijerph18116098 (PMC8200967; doi:10.3390/ijerph18116098)
Supplement: Supplementary file 1 [file ijerph-18-06098-s001.zip › ijerph-1244455-supplementary.pdf]

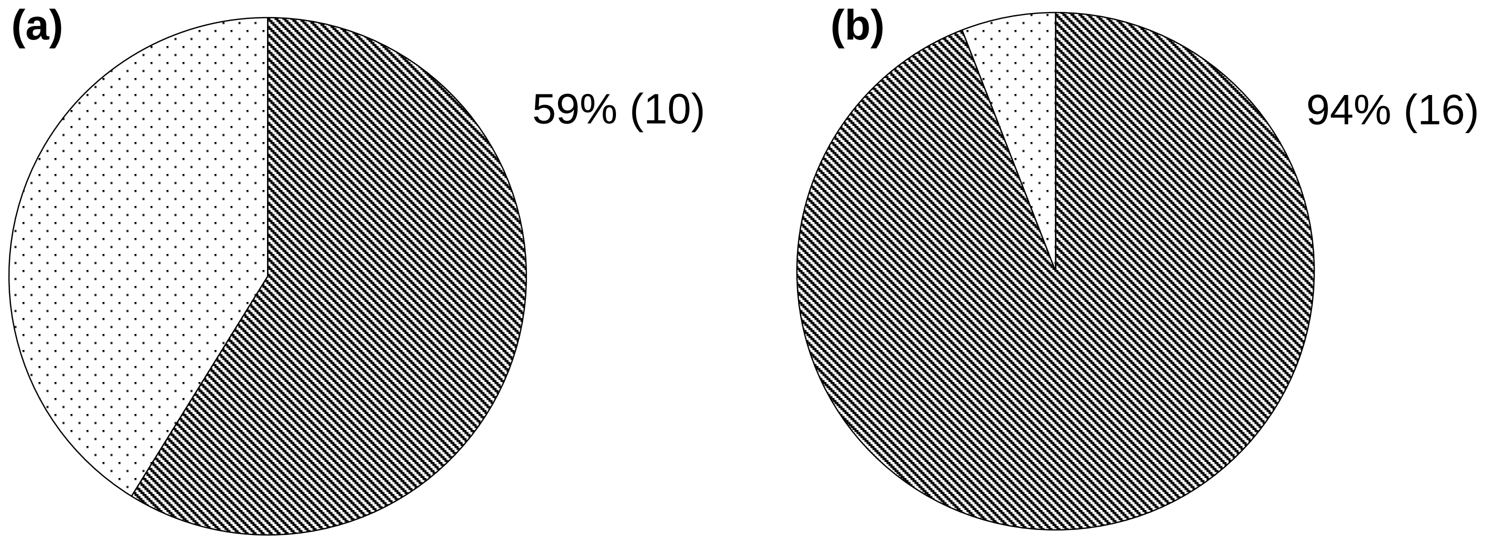

Figure S1. Comparison of effective methods for isolation of thermotolerant campylobacters from water samples: (**a**) ISO standard method and (**b**) novel method. Level of *Campylobacter* detection in positive samples (n = 17) identified by both methods shown in dark colour.

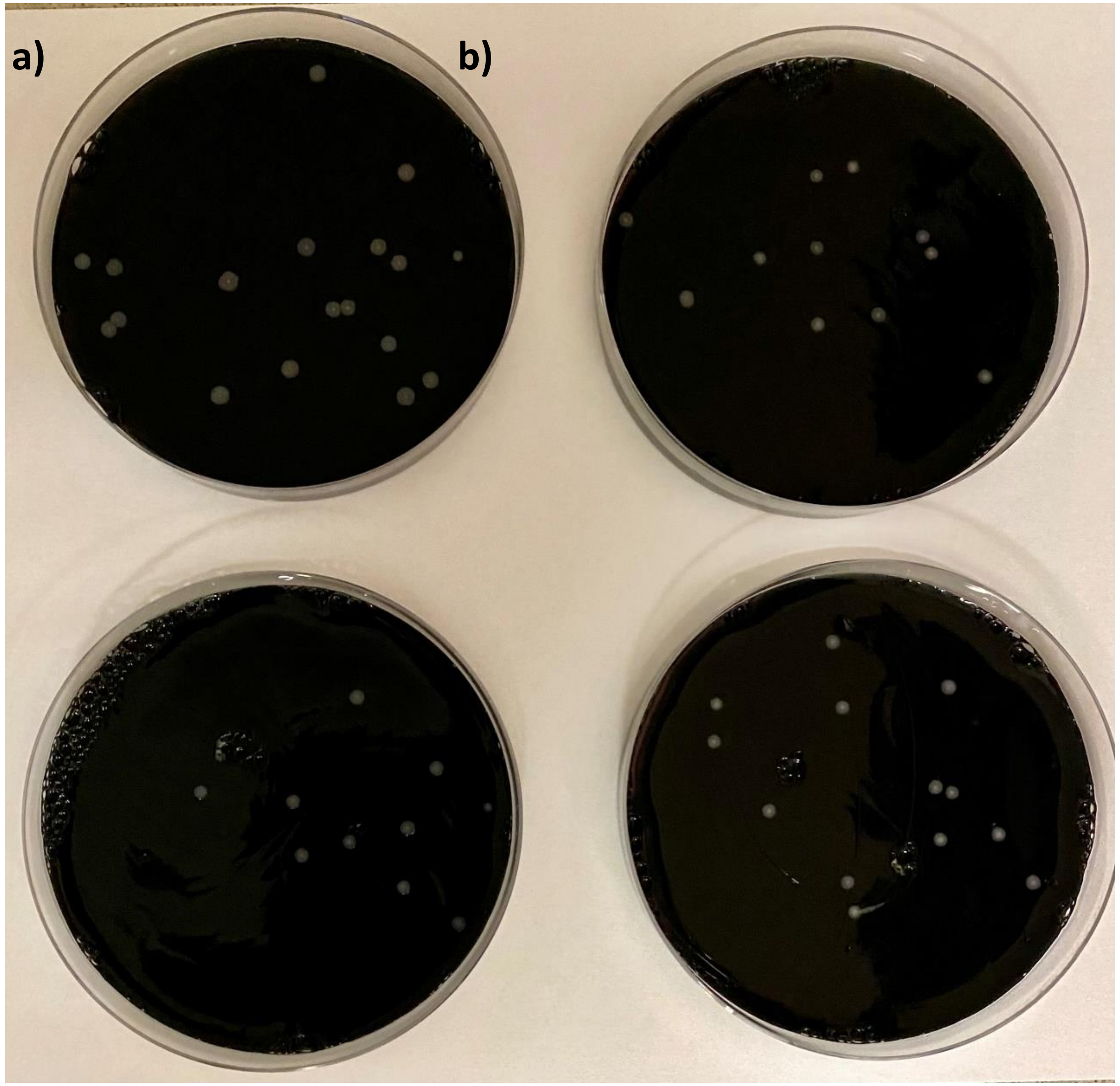

Figure S2. Viability testing of *C. jejuni* and *C. coli* on mCCDA agar (**a**) before and (**b**) after centrifugation.

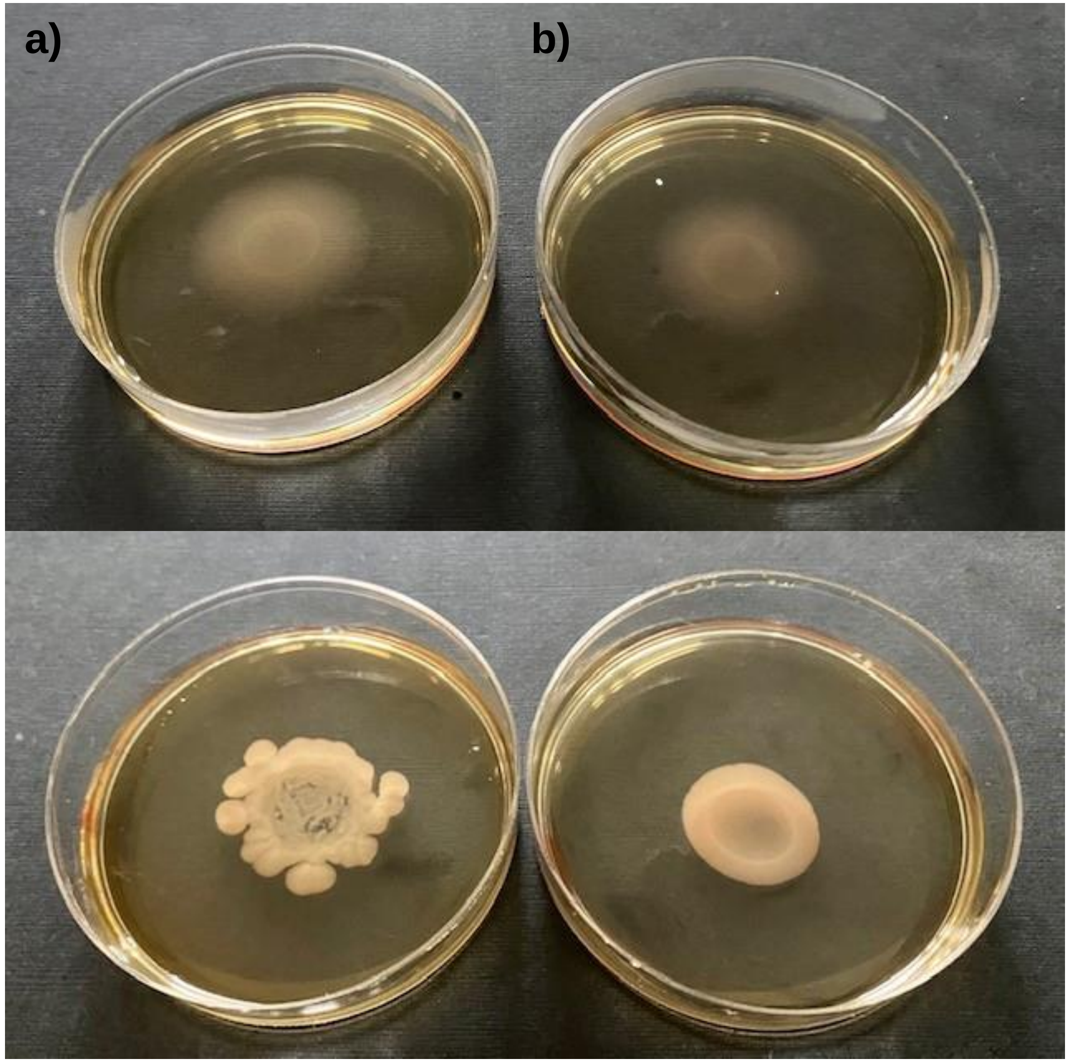

C. coli

K. pneumoniae

Figure S3. *Campylobacter* motility*. C. coli* and non-motile *K. pneumoniae* strains were cultivated on 0.25% agar (**a**) before and (**b**) after centrifugation.

a) b)

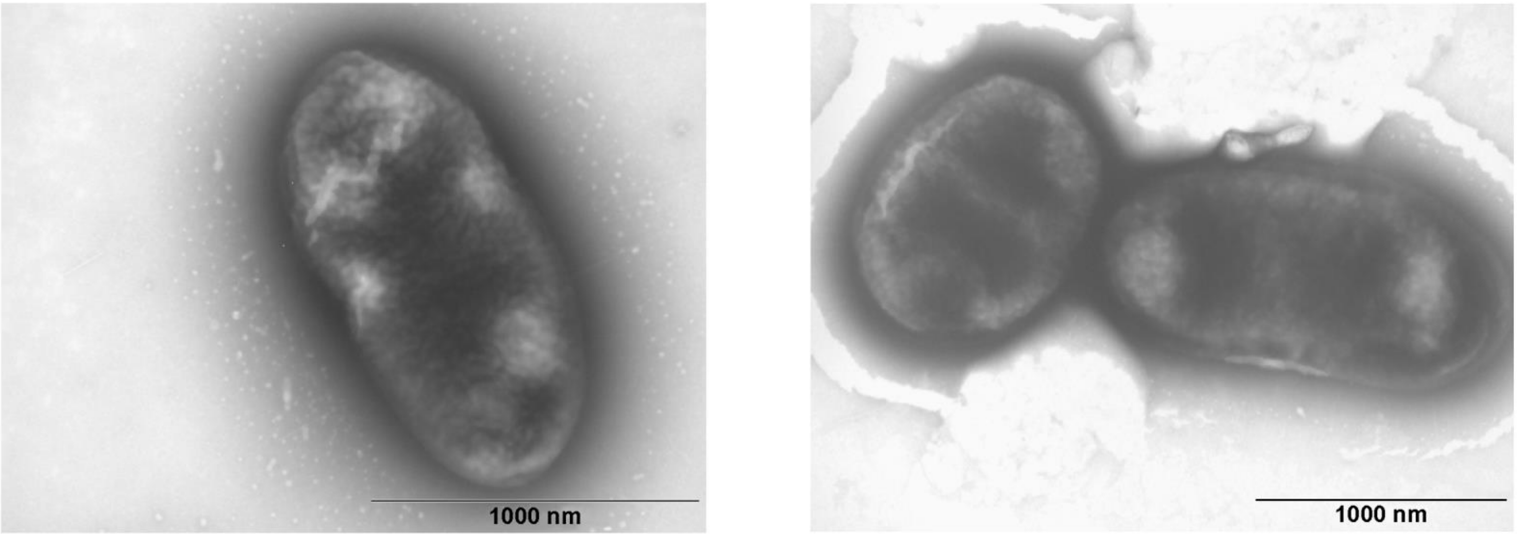

Figure S4. Effect of high-speed centrifugation on *K. pneumoniae c*ell morphology. Representative figures of electron microscopy (**a**) before and (**b**) after centrifugation.

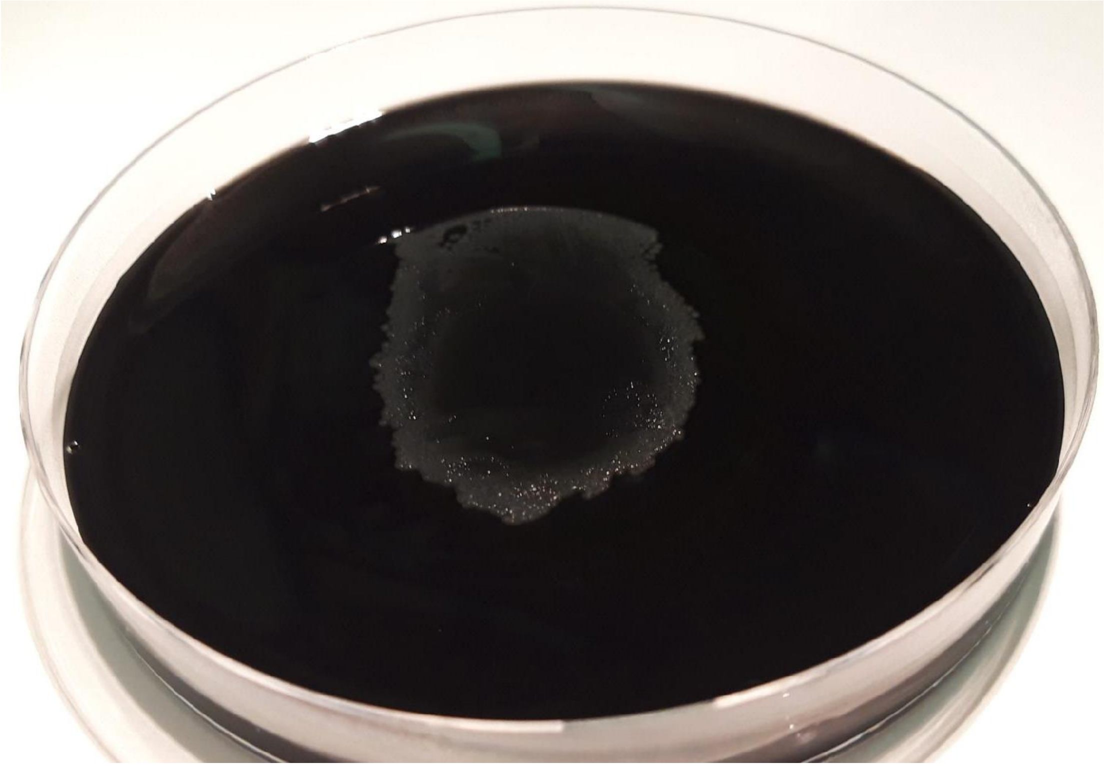

Figure S5. Growth of mixture of thermotolerant *C. jejuni* and *C. coli* with *K. pneumoniae* on mCCDA agar.

a)

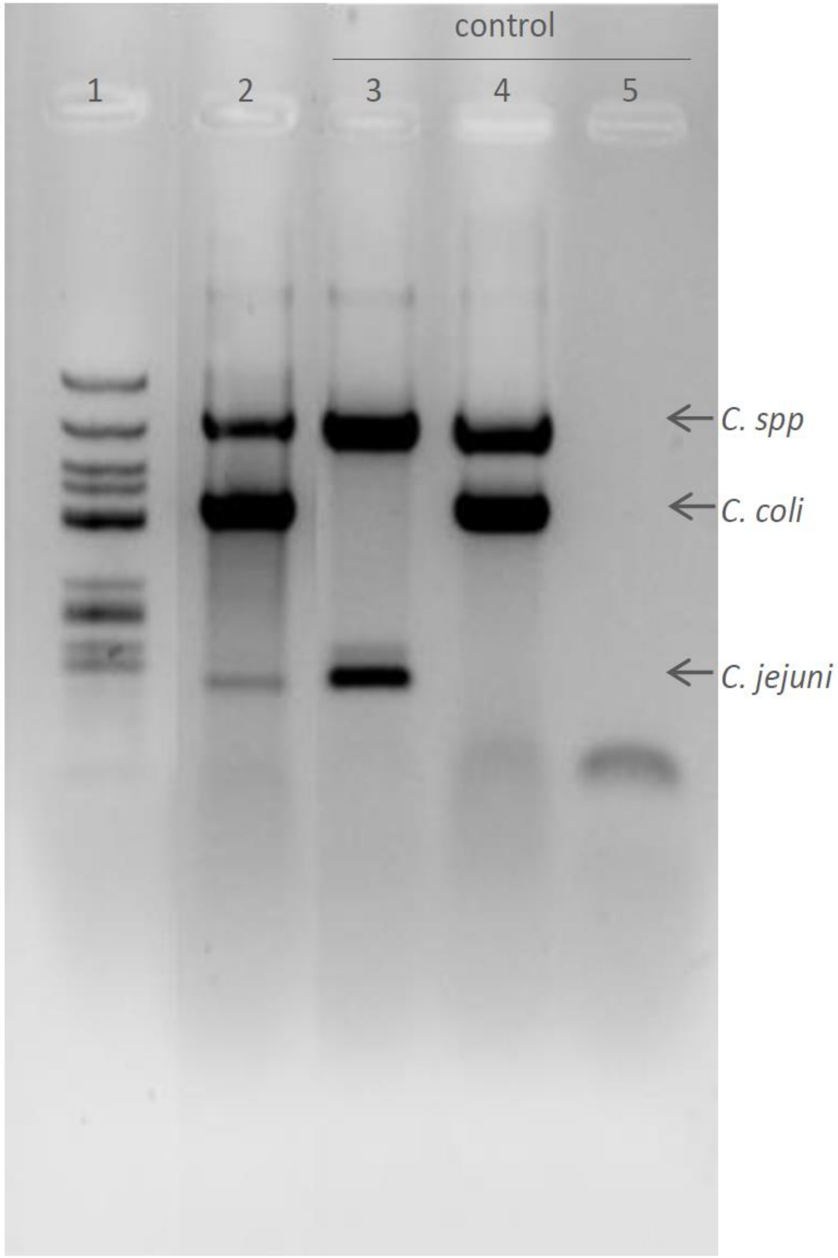

b)

Bruker Daltonik MALDI Biotyper
Classification Results

Project Info:

Project Name:	**2020 10 23 I**
Project Description:	
Project Owner:	Admin@02_MALDI
Project Creation Date/Time:	2020-10-23T11:27:50.549
Project Analyte Count:	20
Project Type:	Development
Validation:	passed
Validation Position:	A15:0

Result Overview

Analyte Name	Analyte ID	Organism (best match)	Score Value	Organism (second best match)	Score Value
A15 (+++) (C)	Standard	Escherichia coli	2.311	Escherichia coli	2.259
B17 (+++) (A)	C5638-CCDA-A	Campylobacter jejuni	2.393	Campylobacter jejuni	2.354
B18 (++) (A)	C5638-CCDA-B	Campylobacter jejuni	2.223	Campylobacter jejuni	2.216
B19 (+++) (A)	C5638-KA-A	Campylobacter jejuni	2.374	Campylobacter jejuni	2.311
B20 (++) (A)	C5638-KA-B	Campylobacter jejuni	2.25	Campylobacter jejuni	2.242
B21 (+++) (A)	C5690-CCDA	Campylobacter coli	2.422	Campylobacter coli	2.247
B22 (+++) (A)	C5690-KA	Campylobacter coli	2.383	Campylobacter coli	2.221
B23 (++) (A)	C5642-CCDA	Campylobacter coli	2.114	Campylobacter coli	2.069
B24 (++) (A)	C5642-KA	Campylobacter coli	2.203	Campylobacter coli	1.92

Meaning of Score Values

Range	Description	Symbols	Color
2.300 ... 3.000	**highly probable species identification**	(+++)	green
2.000 ... 2.299	**secure genus identification, probable species identification**	(++)	green
1.700 ... 1.999	**probable genus identification**	(+)	yellow
0.000 ... 1.699	**not reliable identification**	(-)	red

Meaning of Consistency Categories (A - C)

Category	Description
A	**Species Consistency:** The best match was classified as 'green' (see above). Further 'green' matches are of the same species as the first one. Further 'yellow' matches are at least of the same genus as the first one.
B	**Genus Consistency:** The best match was classified as 'green' or 'yellow' (see above). Further 'green' or 'yellow' matches have at least the same genus as the first one. The conditions of species consistency are not fulfilled.
C	**No Consistency:** Neither species nor genus consistency (Please check for synonyms of names or microbial mixture).

Figure S6. Identification of *C. jejuni* and *C. coli* by PCR and MALDI-TOF/MS after their isolation from mixture of *C. jejuni*, *C. coli* and *K. pneumoniae*. (**a**) PCR: size marker (lane 1), mixture of *C. jejuni*, *C. coli* and *K. pneumoniae* (lane 2), *C. jejuni* positive control (lane 3), *C. coli* positive control (lane 4), negative control (lane 5). (**b**) MALDI-TOF/MS identification.

CC	F: GGTATGATTTCTACAAAGCGAG<br>R: ATAAAAGACTATCGTCGCGTG	500 bp
CJ	F: CAAATAAAGTTAGAGGTAGAATGT<br>R: GGATAAGCACTAGCTAGCTGAT	159 bp
Cspp	F: ATCTAATGGCTTAACCATTA<br>R: GTAACTAGTTTAGTATTCCGG	833 bp

Table S1. Primers used in this study for identification of thermotolerant *C. jejuni* and *C. coli*.

No.	sample ID	method	
		standard	novel
1	BW 180913-27	-	-
2	BP2 180917-28	*C. jejuni*	*C. jejuni*
3	BP1 180917-29	-	-
4	BW 180920-30	*C. coli*	*C. jejuni, C. coli*
5	BP10 180921-31	-	*C. jejuni*
6	BP10 180924-32	*C. jejuni*	*C. jejuni*
7	BW 181010-34	-	*C. coli*
8	BP1 181015-35	-	-
9	BP12 181015-36	-	-
10	BW 181107-42	-	*C. jejuni, C. coli*
11	BW 181205-49	-	*C. jejuni, C. coli*
12	BW 190220-6	-	*C. jejuni, C. coli*
13	BW3 190302-7	-	-
14	BP2 190319-8	-	-
15	BW 190319-9	*C. coli*	*C. jejuni, C. coli*
16	BW 190424-10	*C. coli*	*C. jejuni, C. coli*
17	BP10 190429-11	*C. jejuni*	*C. jejuni, C. coli*
18	BP13 190429-12	-	-
19	BP14 190430-13	-	*C. jejuni, C. coli*
20	BP1 190502-14	-	-
21	BP1 190520-15	-	-
22	BP15 190524-22	-	-
23	BP2 190604-25	*C. jejuni*	-
24	BP16 190610-26	-	-
25	BP17 190610-27	-	-
26	BP18 190617-28	-	-
27	BP19 190617-29	-	-
28	BP20 190625-30	-	-
29	BP21 190625-31	-	-
30	BP22 190625-32	-	-
31	BP23 190626-33	*C. jejuni*	*C. jejuni*
32	BP24 190626-34	-	*C. jejuni*
33	BP25 190626-35	-	-
34	BW 190628-36	*C. jejuni, C. coli*	*C. jejuni, C. coli*
35	BW 190808-45	*C. jejuni, C. coli*	*C. jejuni*
36	BP10 180808-46	-	-

Table S2. List of water samples. BW (waste water), BP (pond/surface water). Locality of ponds from which surface water was collected (1-25). Isolation of *C. jejuni* and *C. coli* strains from mCCDA agar.

A	number of positive samples by both methods	9
B	number of positive samples by standard method	1
C	number of positive samples by modified method	7
D	number of negative samples by both methods	19

diagnostic method	formula	[%]
diagnostic accuracy	(A+D)/(A+B+C+D)x100	77.78
relative sensitivity	A/(A+B)x100	90.00
relative specificity	D/(C+D)x100	73.08

Table S3. Comparison of both methods by diagnostic accuracy [%], relative sensitivity [%], and relative specificity [%].

	dilution 10^5 number of colonies		CFU (x 10^8)		dilution 10^6 number of colonies		CFU (x 10^8)	
	before	after	before	after	before	after	before	after
C. jejuni	123	117	1.23	1.17	18	11	1.80	1.10
C. coli	133	120	1.33	1.20	9	13	0.90	1.30

Table S4. Colony formation of *C. jejuni and C. coli* before and after centrifugation 12 000 g, 30 min.
